# Decarboxylase‐positive *Enterococcus faecium* strains isolated from rabbit meat and their sensitivity to enterocins

**DOI:** 10.1002/fsn3.361

**Published:** 2016-03-29

**Authors:** Andrea Lauková, Renata Szabóová, Pavel Pleva, Leona Buňková, Ľubica Chrastinová

**Affiliations:** ^1^ Institute of Animal PhysiologySlovak Academy of SciencesŠoltésovej 4‐6Košice040 01Slovakia; ^2^ University of Veterinary Medicine and PharmacyKomenského 73Košice041 83Slovakia; ^3^ Department of Environmental Protection Engineering Faculty of TechnologyTomáš Bat'a University in ZlínT.G. Masaryka 5555Zlín760 01Czech Republic; ^4^ Department of NutritionNational Agricultural and Food CentreHlohovecká 2Nitra‐Lužianky7949 41Slovakia

**Keywords:** Decarboxylase, Enterocococci, enterocins, gelatinase, inhibition effect, rabbit meat

## Abstract

**Background:**

The objective of the study was to determine sensitivity of *Enterococcus faecium* strains from rabbit meat to enterocins.

**Results:**

Twenty‐five decarboxylase‐positive strains (from rabbit meat) allotted to the species *E. faecium* by genotypization and by MALDI TOF MS spectrometry identification (evaluation score value range 2.104–2.359; in the range for highly probable species identification‐score value 2.300–3.000 and secure probable species identification/probable species identification‐2.000–2.299) were studied. Seventeen strains were gelatinase positive. Although they did not produce histamine (HIS), spermidine, and spermine, they produce at least one among seven tested biogenic amines (BAs) in small amounts (2–10 mg/L) or up to very high amounts (>1000 mg/L). Putrescine was produced by two strains. These decarboxylase‐positive strains were sensitive to enterocins (Ents). All strains were sensitive to Ent 2019 and Ent 55 (inhibitory activity from 200 to 819 200 AU/mL). Twenty‐two strains were inhibited by Ent A(P) and Ent 4231; 20 strains were sensitive to Ent M.

**Conclusion:**

Our results have spread the basic knowledge related to inhibitory spectrum of enterocins showing sensitivity of decarboxylase‐positive strains to enterocins. Protective possibilities of enterocins in meat processing were also indicated.

## Introduction

Rabbit meat is characterized by high levels of essential amino acids. It is also an important source of highly available micronutrients, such as vitamins and minerals (Dalle Zotte 2004). Safety and shelf life of meat can be limited by microbial growth and contamination. Enterococci belong to the Phylum *Firmicutes*, to the Family *Enterococaccae,* to the genus *Enterococcus* (De Vos et al. 2009). They are generally considered having an ambiguous status with respect to food safety (Ladero et al. 2012). But they also can be considered an asset in, for example, cheese technology since some strains can be used as starter cultures, (Giraffa 2003) others with probiotic properties or those bacteriocin‐producing strains can be used, for example, in rabbits husbandry to prevent and/or eliminate spoilage bacteria (Pogány Simonová et al. 2009; Lauková et al. 2012). Regarding a meat production, lactic acid‐producing enterococci can be found in raw meat, but they can also be associated with processed/fermented meat products (Lauková et al. 2011; Ribeiro et al. 2011) rabbit meat including (Szabóová et al. 2012). The dominant heterogenous microbiota/contaminants on carcasses and packaged rabbit meat are pseudomonads, lactic acid bacteria, yeast or, for example, *Brochothrix thermosphacta* (Rodríguez‐Calleja et al. 2004) with total bacterial counts between 4.01 and 4.96 log CFU/g. Microbial contamination 6.0–7.0 log CFU/g is critical and can lead to spoilage of meat. The slaughtering process may cause extensive contamination of muscle tissue with a vast range of microorganisms. Some of them come from the animal intestinal tract (during evisceration process, especially enteric microorganisms) and others from the environment in contact with the animals before or during slaughter (Hernández and Gondret 2006) enterococci not eliminating (Ribeiro et al. 2011). On the one side, certain enterococcal strains have probiotic properties and they can produce antimicrobial substances‐bacteriocins (Simonová and Lauková 2007; Franz et al. 2011). On the other side, some strains can produce biogenic amines‐BAs (Ladero et al. 2012; Bover‐Cid et al. 2001). BAs and polyamins (cadaverine [CAD], histamine [HIS], phenylethylamine [PEA], putrescine [PUT], tyramine [TYM], spermine [SPN], spermidine [SPD]) are formed from their precursors, amino acids, mainly by decarboxylation activity of microorganisms (Koutsoumanis et al. 2010). Intensity of decarboxylation reaction depends on the presence of particular strains of microorganisms, concentration of free amino acids, and also on many environmental factors such as pH values, presence of oxygen, energy sources, temperature changes, water activity and also the presence of generated BAs (Buňková et al. 2011). The presence of some BAs, especially HIS, PUT, CAD, and TYM can lead to spoilage process in meat (Zhang et al. 2011) and/or to threaten a health of sensitive consumers (Pleva et al. 2012a). Therefore, the ways how to eliminate BAs‐producing enterococci or to prevent their colonization are searched. One very promising possibility is use of bacteriocins. Most bacteriocins produced by enterococci especially by the *Enterococcus faecium* strains are grouped as enterocins (Franz et al. 2007). Interest in bacteriocin study produced by enterococci has been stimulated by the fact that they can inhibit gram‐positive food‐borne pathogens but they are also active against decarboxylase‐positive enterococci. In addition, most of enterocins inhibit Gram‐positive bacteria (Lauková et al. 1993). Because of bacteriocins activity against food‐borne pathogens on one side and consumer demands for more “natural” preservatives on the other side, bacteriocins have been suggested for use as “biopreservatives” (Cleveland et al. 2001). In the study of Franz et al. (2007) a simplified classification scheme is proposed for enterocins, including three classes: Class I enterocins (lantibiotic enterocins), Class II enterocins (small, nonlantibiotic peptides), Class III enterocins (cyclic enterocins), and Class IV enterocins (large proteins). Class II can be subdivided into three subclasses: (II.a) enterocins of the pediocin family; (II.b) enterocins synthesized without a leader peptide, and (II.c) other linear, nonpediocin‐type enterocins. However, Nes, Diep, and Ike (Nes et al. 2014) established a group of bacteriolysins. Many enterocin‐producing *E. faecium* strains were isolated and characterized at our Laboratory of Animal Microbiology, for example, Enterocin A (P) produced by *E. faecium* strain EK13 = CCM 7419 (Lauková et al. 1998a; Mareková et al. 2003). The strain or its enterocin itself were experimentally applied, for example, in tofu cheese to reduce *Listeria innocua* LMG 13568 contamination (Lauková and Mareková 2002). After Ent A(P) application into gnotobiotic Japanese quails infected with *Salmonella enterica* serovar Dusseldorf SA31 were reduced Salmonella cells (Lauková et al. 2003a). Ent M (produced by *E. faecium* AL41 = CCM 8558 (Lauková et al. 1998a; Mareková et al. 2007) showed antilisterial effect during Gombasek sausage processing after its experimental contamination with *L*. *innocua* (Lauková et al. 2003b). Ent 4231 (produced by *E. faecium* CCM 4231, Lauková et al. 1993) showed antagonistic effect against contaminated bacteria in salami Hornad (Lauková et al. 1999b). Ent 2019 (produced by *E. faecium* EF2019 = CCM 7420 (Simonová and Lauková 2007) beneficially influenced microbiota in rabbits husbandry by reducing spoilage bacteria, stimulating phagocytic activity, and by improving morphometry parameters of intestine (Pogány Simonová et al. 2009, 2015). Ent 55 (produced by *E. faecium* EF55 Strompfová and Lauková 2007) reduced coliforms, staphylococci, and enterococci in Japanese quails (Strompfová et al. 2003).

Following the basic research, firstly we were focused on the study of inhibitory activity of our enterocins against decarboxylase‐positive enterococci from rabbit meat; secondly, to follow consumers safety, a new way how to prevent/eliminate decarboxylase‐positive microbiota (enterococci) was indicated.

## Material and Methods

### Isolation and identification of enterococci

Enterococci were isolated from back limbs (*Musculus biceps femoris*) of 42 healthy farming rabbits‐breed Hyplus, aged 56 days (farm at National Agricultural and Food Centre, Nitra‐Lužianky, Slovakia). Sampling and all animal care followed the Guide for the Care of the Animals accepted by the Ethic Commission and by the Slovak Veterinary and Food Administration. Samples (10 g) from back limb of individual rabbits were put into 90 mL of buffered peptone water (pH 7.0 ± 0.2, Biomark Lab., Dalviwadi, Dhairi Pune, India), homogenizate by the Stomacher (Masticator, IUL Instruments, Spain) and diluted by the standard microbiological method (ISO, International Organization of Standardization, ratio 1:9) as previously described by Szabóová et al. (2012). The volume (100 *μ*L) of the appropriate dilution was plated onto Kanamycin Esculin Agar (ISO‐7899‐2, KEA; Becton Dickinson, Cockeysville, USA) and incubated at 37°C for 24 h in a 5% gaseous CO_2_ – air. The colonies grown on KEA were randomly picked, checked for purity and maintained on M‐Enterococcus agar plates (Difco Lab., Detroit, Maryland, USA). Thirty‐four typical colonies were genotyped using PCR‐Techgene KRD thermocycler (Techne, United Kingdom) described previously by Szabóová et al. (2012) and Pleva et al. (2012b). The PCR products were visualized (10 *μ*L of each) using the electrophoresis in 0.8% agarose gels (Sigma Aldrich, Hamburg, Germany) buffered with 1xTAE (Merck, Darmstadt, Germany) containing 1 *μ*g/mL ethidium bromide (Sigma, Germany). The molecular mass standard (Promega Corp., Madison, Wisconsin, USA) was used according to the manufacturers instructions. Moreover, presumed colonies were identified using the MALDI‐TOF MS (Mass spectrometry) based on protein “fingerprints” (MALDI‐TOF MS, Bruker Daltonics Alatoom et al. 2011), performed using a Microflex MALDI‐TOF MS mass spectrophotometer. Briefly, a single colony from M‐Enterococcus agar was mixed with matrix (*α*‐cyano‐4‐hydroxycinnamic acid and trifluoroacetic acid) and the suspension was spotted onto a MALDI plate and ionized with a nitrogen laser (wave‐length 337 nm, frequency 20 Hz). The results were evaluated using the MALDI Biotyper 3.0 (Bruker Daltonics, Billerica, Maryland, USA) identification database. Taxonomic allocation was evaluated on the basis of highly probable species identification (value score 2.300–3.000) and secure probable species identification/probable species identification (2.000–2.299). Positive controls were those involved in the identification system. Identical colonies (9) evaluated by MALDI‐TOF MS score value were excluded. The target of 25 *E. faecium* strains were tested for gelatinase phenotype and decarboxylase activity.

### Gelatinase phenotype and determination of biogenic amines

Gelatinase is a toxin, extracellular metalloendopeptidase which hydrolysis gelatin, collagen, hemoglobin, etc., and belong to virulence factors causing infections. Gelatinase phenotype was testing as follows: Todd‐Hewitt agar with 3% gelatin (Becton and Dickinson, Cockeysville, USA) was used for gelatinase testing. Loss of turbidity‐halos around colonies was checked after strain growth (48 h at 37°C) and flooding the plates with a 15% solution (HgCl_2_ in 20% HCl).

To determine BAs, enterococci were cultivated in the de Man–Rogose–Sharpe broth (HiMedia, India; MRS) enriched with precursors of the tested BAs (2 g/L each amino acids: histidine, tyrosine, arginine, ornithine, and lysine, Sigma‐Aldrich, St. Louis, Montana, USA). The cultivation was carried out at 30 ± 1°C for 24 h. Each isolate was cultivated five times according to Buňková et al. (2009a). The production of seven BAs (CAD; HIS; PEA; PUT; TYM; SPD; SPN) was monitored by a high‐performance liquid chromatography system equipped with a binary pump; an autosampler (LabAlliance, Ramsey, Minnesota, USA); a column thermostat; a UV/VIS DAD detector (*λ* = 254 nm); and a degasser (1260 Infinity, Agilent Technologies, Washington, USA). After cultivation of the tested bacteria, the broth was centrifuged (4000 *g*; 22 ± 1°C; 20 min) and the acquired supernatant was diluted (1:1; v/v) with perchloric acid (c = 0.6 mol/L). The mixture was filtered (porosity 0.22 *μ*m) and the acquired filtrate was subjected to derivatization with dansylchloride according to Dadáková et al. (2009); 1,7‐heptanediamine was used as an internal standard. The derivatized samples were filtered (porosity 0.22 *μ*m) and applied on a column (Cogent Column HPS C18, 150 × 4.6 mm, 5 *μ*m). The conditions for separation of the monitored BA are described by Smelá et al. (2004). Each of the five cultivated broths (for one tested microorganism) was derivatized twice and each derivatized mixture was applied twice on the column (*n* = 20). Based on the data analysis, the tested microorganisms were classified into five groups according to their decarboxylation activity: BAs not detected or lower than 2 mg/L (<2 mg/L; −); low production (2–10 mg/L; +); medium production (10–100 mg/L; ++); high production (100–1000 mg/L; +++); and very high production (>1000 mg/L; ++++) previously reported by Pleva et al. (2012b).

### Enterocins preparation, testing sensitivity of enterococci to enterocins

Five partially purified enterocins (10 *μ*L of each) were used produced by our *E. faecium* strains of different origin (four deponed to Czech Culture of Microorganisms in Brno, Czech Republic): Ent A (P) (produced by environmental strain *E. faecium* EK13 = CCM 7419 – Mareková et al. 2003); Ent M (produced by *E. faecium* AL41 = CCM 8558, environmental strain – Lauková et al. 1998a; Mareková et al. 2007) Ent 4231 (produced by ruminal strain *E. faecium* CCM 4231 – Lauková et al. 1993) Ent 2019 (produced by rabbit‐derived strain *E. faecium* EF2019 = CCM 7420 – Simonová and Lauková 2007); Ent 55 (produced by *E. faecium* EF55 isolated from chicken – Strompfová and Lauková 2007). Enterocins were prepared by the following procedure: a 16 h culture (300 mL of *E. faecium* EK13 = CCM 7419, EF2019 = CCM 7420, EF55, CCM4231, EF AL41 = CCM 8558 strains in MRS broth, Merck, Germany) were centrifuged for 30 min at 10,000 *g* in order to remove the cells. After adjusting of supernatant to pH 5.0 (5.5 in the case of AL41 = CCM8558 strain), ammonium sulfate was gently added to the supernatants to obtain 40% (w/v) saturation. The mixture was stirred at 4°C for 2 h (EK13 = CCM 7419), for 4 h (EF2019 = CCM 7420), and for 24 h (EF55, CCM 4231), at 21°C for 1 h (AL41 = CCM 8558). After centrifugation at 10,000 *g* for 30 min, the resulting pellet was resuspended in 10 mmol/L of sodium phosphate buffer (pH 6.5). Their inhibitory activity was tested by the agar spot test (De Vuyst et al. 1996) against the principal indicator –*E. avium* EA5 (inhibitory activity reached up to 819,200 AU/mL). Inhibitory activity is expressed in AU per mL, it means the reciprocal of the highest twofold dilution of Ent demonstrating complete growth inhibition of the indicator strain.

The target of 25 decarboxylase‐positive strains isolated from rabbit meat was used as indicator strains and tested for their sensitivity to enterocins (De Vuyst et al. 1996). Briefly, Brain‐heart infusion (BHIA) supplemented with 1.5% agar (Becton Dickinson, USA) was used for the bottom agar layer. For overlay, 0.7% BHIA enriched with 200 *μ*L of a 18 h culture of indicator strain (grown up to an average optical density‐OD_600_ = 0.800 nm) was used. On the surface of soft agar with tested strain, 10 *μ*L of each Ent (diluted in phosphate buffer, pH 6.5) was dropped. The plates were incubated at 37°C for 18 h. Clear inhibitory zones around drops of Ent were checked and the antimicrobial (inhibitory) activity was evaluated in AU/mL. The tests were repeated twice.

## Results and Discussion

Thirty‐four enterococcal strains from 42 back limbs of broiler rabbits were allotted to the species *E. faecium* (by PCR as previously documented by Szabóová et al. 2012). Moreover, followed the MALDI‐TOF MS mass spectrometry, 25 strains were confirmed as *E. faecium* with an evaluation score value in the range 2.104–2.359, which is in the range for highly probable species identification (value score 2.300–3.000) and secure probable species identification/probable species identification (2.000 – 2.299 Alatoom et al. 2011). Among those 34 strains identified by PCR, nine colonies were excluded as identical following the MALDI‐TOF MS identification. The species *E. faecium* represents the most frequently detected ubiquitous microorganism which constitute a large proportion of autochthonous microflora in the gastrointestinal tract of farm animal, humans but also in food sources as is also meat (Foulquié Moreno et al. 2006; Rehaiem et al. 2014). The strains detected could have their origin, for example, in slaughtering process and/or manipulating with the animal in the slaughterhouse. Most strains were gelatinase positive (17, Table 1) by phenotype testing. Eight strains were gelatinase negative. Gelatinase activity is one among virulence factors which can cause infections, although here gelatinase gene detection was not provided. The ability to produce gelatinase, this extracelullar zinc‐endopeptidase capable of hydrolyzing gelatin, collagen, casein, and other small biologically active peptides can be explained easily because of the usual presence of such components in the meat produces meat products (Ribeiro et al. 2011). Twenty‐five strains tested were evaluated as BAs‐producing microorganisms. In spite of the fact that they did not produce HIS, SPD and SPN, they produce at least one BA, for example, *E. faecium* M1C, M7b, M6C strains produce CAD, *E. faecium* M2cB, M4aB produce TYM, although only low amount (up to 10 mg/L). On the other side, the strains EFM4C, M2cA produce only TYM but in very high amount (>1000 mg/L). Two strains among 25 were found to produce PUT in high amount (EFM2C and EFM3b (577,622 mg/L) and these strains produce also CAD and TYM. PEA is produced by six strains (Table 1) in low amount (up to 10 mg/L) and medium amount (up to 100 mg/L). Twenty‐two strains produce TYM in the range from very low to very high level (Table 1; 2–3 738 mg/L) and 21 strains produce CAD, however, only in low amount (3.8–8.9 mg/L). Nine strains produced two BAs (in each) and 7 only one. The presence of BAs is usually connected with food poisoning and can thereby threaten health of consumers (Silla Santos 1996). Therefore, to know sensitivity of decarboxylase‐positive strains to antimicrobials, for example, to enterocins is promising way how to treat “contaminated” food.

**Table 1:** Gelatinase activity and decarboxylase activity of tested enterococci (production of biogenic amines)

	Gel	CAD	TYM	PEA	PUT
EF M1C	−	+	−	−	−
EF M2C	−	+	+	−	+++
EF M3b	+	+	++	−	+++
EF M7b	−	+	−	−	−
EF M4C	+	−	++++	−	−
EF M6C	+	+	−	−	−
EF M5a	+	+	+	+	
EF 5BM1	−	+	++++	++	−
EF M2a	+	+	+	−	−
EF M2cA	+	−	++++	−	−
EF M2cB	−	−	+	−	−
EF M4aA	+	+	++++	++	−
EF M4aB	+	−	+	−	−
EF M5aA	+	+	+	+	−
EF M5aB	−	+	++++	++	−
EF M7Ba	−	+	+	−	−
EF 1BM	+	+	+	−	−
EF.M3A	+	+	+	−	−
EF 4BM1	+	+	+	−	−
EF M1B	+	+	++++	++	−
EF M2c	−	+	+	+	−
EF M1c	+	+	+	−	−
EF M6c	+	+	+	−	−
EF M4B	+	+	+	−	−
EF M2A	+	+	+	−	−

Gelatinase + (positive), (<2 mg/L; −) very low production of biogenic amines or biogenic amines not detected; low production (2–10 mg/L; +); medium production (10–100 mg/L; ++); high production (100–1000 mg/L; +++); very high production (>1000 mg/L; ++++). Histamine, spermine, and spermidine were not produced by the tested strains.

CAD, cadaverine; TYM, tyramine; PEA, phenylethylamine; PUT, putrescine.

Most of our decarboxylase‐positive enterococci were sensitive to antimicrobial substances‐enterocins (Ents, Table 2). All strains were sensitive to Ent 2019 (produced by rabbit‐derived strain) and Ent 55 (from poultry) with inhibitory activity from 200 to 819 200 AU/mL. Twenty‐two strains were inhibited by Ent A(P) and Ent 4231 and 20 strains were sensitive to Ent M (Table 2). On the other hand, the least sensitive was *E. faecium* M5aB; it was resistant to Ent A(P), Ent M, and Ent 4231. All strains were sensitive to Ent 2019 and Ent 55 reaching activity in the range from 200 to 819 200 AU/mL (Table 2). Using Ent 2019 the highest activity in AU/mL was measured; almost the same as was its initial activity, for example, *E. faecium* EFM3b, EFM4aB, and EFM1B were inhibited by the activity 409 600 AU/mL; *E. faecium* M2C, M7C, M6C, and EFM5a were inhibited by the activity 819 200 AU/mL. *E. faecium* M5aA and 4BM1 were not inhibited by Ent A(P) and Ent M. Simonová and Lauková (2007) reported the inhibitory activity of Ent 2019 against enterococci from different sources, for example, from dogs, feces of rabbits with activity 6400 AU/mL as well as against the principal indicator strain *E. faecium* EA5 (3200 AU/mL). Ent 2019 inhibited the growth of *Clostridium*‐like, *Pseudomonas*‐like bacteria and *E. coli*, although lower values of AU were measured (100–200 AU/mL). Moreover, under in vivo conditions Ent M reduces coliforms in rabbits and stimulates phagocytic activity (Lauková et al. 2012). Bacteriocins synthesized by lactic acid‐producing bacteria are known to have the inhibitory effects toward different microbiota (Klaenhammer 1993). The effect of bacteriocins, enterocins including can be influenced by many factors such as purity of bacteriocin, dose, concentration, environment, sensitivity of the strain or resistance genes, etc. Héchard and Sahl (2002) reported the inhibitory activity of bacteriocins of Gram‐positive bacteria based on the interaction with membrane of sensitive cells (bactericidal effect on the formation of pores in the cell membrane). Many bacteriocins of Class II play role to weak and dissipate the movement of protons (PMF – proton motive force) of the target cells through the pore formation by the production of the anion or cation‐specific pores. Lauková et al. (1993) described as first in vitro inhibitory activity of Ent 4231 related to animal origin enterococci; the growth of staphylococci, enterococci, and *E. coli* EC5 was inhibited and the indicator strain *S. aureus* Oxford 209P was inhibited by the highest activity (inhibitory zone more than 9 mm). Antimicrobial effect of Ent 4231 with activity 3 200 AU/mL was also noted after its application in slurry; there the significant reduction in *E. coli*,* Pseudomonas*‐like sp., and enterococci was observed (Lauková et al. 1998b). Ent 4231 (3 200 AU/mL) also inhibited the growth of *S. aureus* SA1 and showed antilisterial effect in dairy products (Lauková et al. 1999a, 2001). Similarly, Strompfová et al. (2003) reported the antimicrobial activity of Ent 55 against the principal indicator *E. faecium* EA5 3 200 AU/mL); moreover, the reduction in enterococci, staphylococci, micrococci, lactobacilli, and lactococci was detected. Belguesmia et al. (2010) reported the effect of enterocin S37, produced by *E. faecalis* S37 (isolated from feces of chicken) against Gram‐positive bacteria, such as *L. monocytogenes*,* L. innocua*,* E. faecalis* JH2‐2, and *Lactobacillus brevis* F145.

**Table 2:** Sensitivity of decarboxylase‐positive strains (biogenic amines‐producing strains) to enterocins

Decarboxylase‐positive strains	A (P)	M	4231	2019	55
EF M1C	100	100	100	51,200	800
EF M2C	3200	100	100	819,200	1600
EF M3b	1600	100	400	409,600	1600
EFM7b	6400	ni	ni	819,200	3200
EF M4C	800	100	100	51,200	800
EF M6C	3200	100	1600	819,200	3200
EF M5a	1600	100	1600	819,200	1600
EF 5BM1	100	100	400	51,200	1600
EF M2a	800	100	200	51,200	1600
EF M2cA	400	100	200	51,200	1600
EF M2cB	800	100	100	51,200	800
EF M4aA	100	100	400	51,200	800
EF M4aB	12,800	100	400	409,600	1600
EF M5aA	ni	ni	200	25,600	100
EF M7Ba	800	100	100	51,200	800
EF 1BM	400	100	100	51,200	800
EF M3A	1600	100	200	51,200	400
EF 4BM1	ni	ni	800	51,200	200
EF M1B	3200	100	800	409,600	3200
EF M2c	1600	100	100	51,200	800
EF M1c	1600	100	100	51,200	800
E.sp.M5a	3200	ni	ni	51,200	400
EF M6c	1600	100	100	51,200	800
EF M4B	3200	100	400	51,200	800
EF M2A	3200	100	200	51,200	400

Initial activity of Enterocins ranged from 200 to 819 200 AU m/L (Arbitrary unit per milliliter).

ni‐not inhibited.

## Conclusion

Twenty‐five mostly gelatinase positive and decarboxylase‐positive enterococci were found to be sensitive to antimicrobial substances‐enterocins. In spite of the fact that they did not produce HIS, SPD, and SPN, they produce at least one of seven tested BAs. Our results spread the basic knowledge related to inhibitory spectrum of enterocins showing sensitivity of decarboxylase‐positive strains to enterocins and indicate protective possibilities of enterocins in meat processing.

## Conflict of Interest

None declared.
